# Simultaneous cytomorphological and multiparameter flow cytometric analysis of ALK-positive anaplastic large cell lymphoma in children

**DOI:** 10.3892/ol.2012.1034

**Published:** 2012-11-19

**Authors:** HONGQIANG SHEN, YONGMIN TANG, XIAOJUN XU, HONGFENG TANG, WEIZHONG GU

**Affiliations:** 1 Division of Hematology-Oncology, Key Laboratory of Reproductive Genetics (Zhejiang University), Ministry of Education, Hangzhou 310003, P.R. China; 2 Division of Pathology, Children’s Hospital of Zhejiang University School of Medicine, Hangzhou 310003, P.R. China

**Keywords:** flow cytometry, anaplastic large cell lymphoma, diagnosis, anaplastic lymphoma kinase

## Abstract

The aim of this study was to investigate the pathological features of anaplastic lymphoma kinase (ALK)-positive anaplastic large cell lymphoma (ALCL) in children and to establish the effectiveness of screening and diagnosing ALCL with multiparameter flow cytometry immunophenotyping (FCI) of lymphoid tissue samples. A total of 121 lymph node tissue specimens obtained from 121 patients with a suspected diagnosis of lymphoma were analyzed with cytomorphological and FCI analysis. Fifteen cases were diagnosed as ALK-positive ALCL based on the pathological features and immunohistochemical results. Of these, there were 3 different types, common type (10 cases), lymphohistiocytic type (4 cases) and neutrophil-rich type (1 case). Thirteen cases (10 common, 2 lymphohistiocytic and 1 neutrophil-rich type) were diagnosed as ALCL using FCI. These cases were CD30-positive and aberrantly expressed at least two T-cell antigens, including CD4 (84.6%), CD2 (76.9%), CD7 (61.5%), CD3 (53.8%) and CD5 (38.4%). Neoplastic cells accounted for only a small proportion of the total cells in FCI, with a median of 19.3% (range, 7.9–31.8%), which was significantly higher than those in the control groups (all <1.0%). The sensitivity of FCI for diagnosing ALCL in lymph node samples was 86.7% with a specificity of 100%. The majority of neoplastic cells demonstrated high light forward and high light side scatter, similar to monocytes or granulocytes in dot plots. FCI may be used as an adjunct to histopathological examination for rapid and reliable diagnosis of pediatric ALCL. Flexible gating strategies and careful analysis are required to identify neoplastic cells with FCI.

## Introduction

Anaplastic large cell lymphoma (ALCL) is a peripheral T-cell-derived malignancy, representing 2–3% of all lymphoid neoplasms, according to estimates by the World Health Organization (WHO) (1). It was first recognized by Stein *et al*(2) in 1985, who reported the consistent expression of the Ki-1 antigen (later designated as CD30) in tumors with frequent cohesive proliferation of large pleomorphic cells. Two different types are recognized as systemic forms, the anaplastic lymphoma kinase (ALK)-positive and ALK-negative ALCL. Regarding genetic and clinical features, the former is characterized by the deregulated expression of chimeric proteins expressing the intracytoplasmic domain of the ALK gene, with a strong and uniform expression of CD30 and ALK proteins (3). Since ALK(−) ALCL lacks distinctive immunophenotypic features and appears to have a prognosis similar to peripheral T-cell lymphoma not otherwise specified (PTCL-NOS), it should be considered a subtype of PTCL-NOS (4–6). Previously, it was suggested that ALK(−) ALCL should continue to be separated from ALK(+) ALCL (6). In the WHO classification, ALK(−) ALCL was regarded only as a provisional entity (1).

Evaluation of suspected tissue by histopathologists is crucial for the diagnosis and accurate classification of ALCL. Flow cytometric immunophenotyping (FCI) is widely performed on bone marrow aspirates and peripheral blood specimens, and is commonly used to diagnose and classify leukemias, myeloproliferative disorders and autoimmune lymphoproliferative disorders as well as to assess residual disease in leukemias. Combined use of cytology and FCI analysis of fine needle aspiration (FNA) samples has been found to contribute to an improved classification of non-Hodgkin lymphomas (NHL) into WHO categories (7,8). However, the majority of previous studies on diagnosing lymphoma with FCI have focused primarily on common T- or B-cell NHL. Since ALCL is a distinct subtype of lymphoma and ALK expression is not available for a considerable subset of the cases analyzed, only a limited number of reports studying the role of FCI in tissue diagnosis of ALCL are available (9,10). In the present study, we reviewed the FCI and morphology results of lymph node biopsy specimens from 15 patients with ALK(+) ALCL to investigate its pathological features in children and to evaluate the role of flow cytometry in diagnosing ALK(+) ALCL.

## Materials and methods

### Subject selection

Lymph node tissues obtained from biopsy specimens of patients with a suspected diagnosis of lymphoma were studied. The specimens were obtained from the main surgical centers in the Children’s Hospital of Zhejiang University School of Medicine, China, from January 2004 to March 2012. Each sample was divided in two; one fresh sample was sent to the Hematology-Oncology Laboratory for FCI analysis and the other to the Department of Pathology for cytomorphological analysis. Samples with insufficient material for the two diagnostic processes were excluded and sent solely to the histopathology department.

A total of 121 specimens were analyzed with FCI and cytomorphological analysis. According to the pathological features and immunohistochemical results, samples were definitively diagnosed as follows: 27 patients with reactive hyperplasia disease; 4 patients with tuberculosis; 11 patients with necrotic lymphadenitis; 7 patients with Hodgkin lymphoma (HD); 25 patients with B-NHL; 32 patients with T-lymphoblastic lymphoma (LBL) and 15 patients with ALCL. The patients with ALCL consisted of 10 males and 5 females with a male to female ratio of 2:1. The median age of this cohort was 10.3 years (range 2.0–15.0). All 15 ALCL patients presented varying degrees of lymphadenopathy at diagnosis. Ten cases had systemic disease (involving ≥2 lymph nodes or extranodal areas, most commonly in the liver, spleen and mediastinum) and 5 cases had the disease limited to ≤1 lymph node with a diameter of 2–6 cm. Nine patients presented fever and 2 of these experienced pleural effusions. The median follow-up time was 17 months (range, 6 months to 3 years) and 3 patients succumbed during this period.

### Pathological and immunohistochemical analysis

The lymph node biopsy specimens were fixed in 10% neutral-buffered formalin and sent to the pathology laboratory immediately. Once the samples were dehydrated, they were embedded in paraffin. Unstained 4 *μ*m sections were cut from each tissue block. Hematoxylin and eosin (H&E), periodic acid-Schiff (PAS) and immunohistochemical staining were carried out for the histopathological study. Macroscopic and microscopic examinations were performed by at least two experienced pathologists. Sections were stained immunohistochemically using the ChemMate™ Dako EnVision™/horseradish peroxidase (HRP) two-step system (DakoCytomation, Glostrup, Denmark). Antibodies were used to detect the expression of CD1a, CD3, CD20, CD43, CD79a, CD30, CD15, CD68, ALK, epithelial membrane antigen (EMA) and Epstein-Barr virus (EBV). Antibodies were obtained from DakoCytomation (Denmark). Appropriate positive and negative controls were set.

### FCI analysis

FCI and histopathological studies were performed separately and in a blinded manner. Fresh biopsy tissue samples were sliced and disaggregated through a mesh <100 *μ*m and the cells were suspended in phosphate-buffered saline (PBS) containing 0.1% sodium azide. Specimens were analyzed for various antigens using a flow cytometer (FACSCalibur™, Becton Dickinson, San Jose, CA, USA). Data acquisition and analysis were performed using CellQuest software (Becton Dickinson). A minimum of 10,000 events were acquired for analysis. Immunophenotyping determination was performed by four-color immunofluorescent staining using the commercially available fluorescently-labeled monoclonal antibodies: mouse IgG1, mouse IgG2a, CD1a, CD2, CD3, CD4, CD5, CD7, CD8, CD10, CD11c, CD19, CD20, CD22, CD23, CD25, CD30, CD34, CD38, CD45, CD56, CD69, CD103, CD138, human leukocyte antigen (HLA)-DR, CD45RA, CD45RO, FMC7, T cell receptor (TCR) α/β, TCRγ/δ, C*μ*, SmIgM, κ and λ (Becton Dickinson). In particular, CD1a, CD2, CD3, CD4, CD5, CD7, CD8, CD25, CD30, CD45, CD69, CD56, TCRα/β, TCRγ/δ, CD45RA and CD45RO were used for T-cell lymphoproliferative disease, while CD10, CD11c, CD19, CD20, CD22, CD23, CD34, CD38, CD103, CD138, FMC7, HLA-DR, C*μ*, SmIgM and surface κ and λ light chains were used for B-cell lymphoproliferative disease.

### Statistical analysis

The diagnostic sensitivity and specificity of FCI were calculated by the Chi-square test. Comparison of the mean flourescence intensity (MFI) of antigen expressions between normal and neoplastic cells was performed by the Wilcoxon signed-rank test. Statistical analyses were performed using the Statistical Package for the Social Sciences (SPSS) software, version 12.0 (SPSS Inc., Chicago, IL, USA). P<0.05 was considered to indicate a statistically significant difference.

## Results

### Pathological findings

According to the pathological features and immunohistochemical results, and with reference to the new WHO classification, 15 cases were diagnosed as ALK(+) ALCL. Of these, 10 were common type, 4 were lymphohistiocytic type and 1 was neutrophil-rich type. Their morphological variants and immunohistochemical results are shown in Table I.

**Figure 1.**
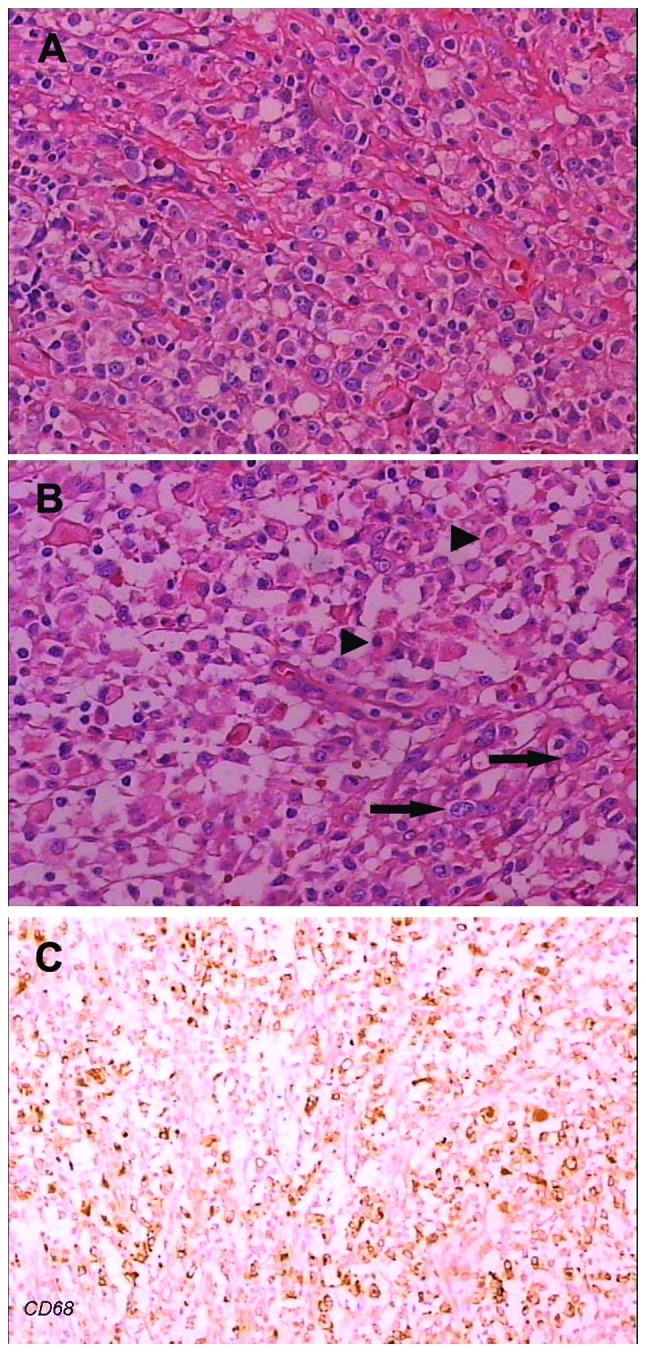
Morphological and immunophenotypical features of ALCL. (A) ALCL, common variant (case 6). Paraffin section shows an area with a predominant population of large neoplastic cells with irregular nuclei and scattered hallmark cells (hematoxylin and eosin staining, H&E; original magnification, ×200). (B) ALCL, lymphohistiocytic variant (case 14). Medium- to large-sized neoplastic cells (arrow) are admixed with reactive histiocytes (arrowhead). Necrosis was evident in the background (H&E; original magnification, ×200). (C) Immunoreactivity of the same case as in (B) with the CD68 antibody. Histiocytes are positive for CD68. ALCL, anaplastic large cell lymphoma.

In the 10 cases with common type ALCL, paraffin sections revealed that the lymph node structure had been destroyed completely or partially with a diffuse proliferation of neoplastic cells and fibrosis. Neoplastic cells were polymorphous with irregular nuclei (horseshoe, folded and multinucleated) and compartmentalized into nests by thin bands of fibrous tissue. Multinucleated cells with Reed-Sternberg-like appearance were observed (Fig. 1A). In the 4 cases with lymphohistiocytic-type ALCL, paraffin sections revealed that the polymorphous neoplastic cells dispersed but did not form nests. A number of mature lymphocytes and histiocytes were evident in the background. In 2 cases, necrosis and fewer neoplastic cells were observed (Fig. 1B and C).

**Figure 2.**
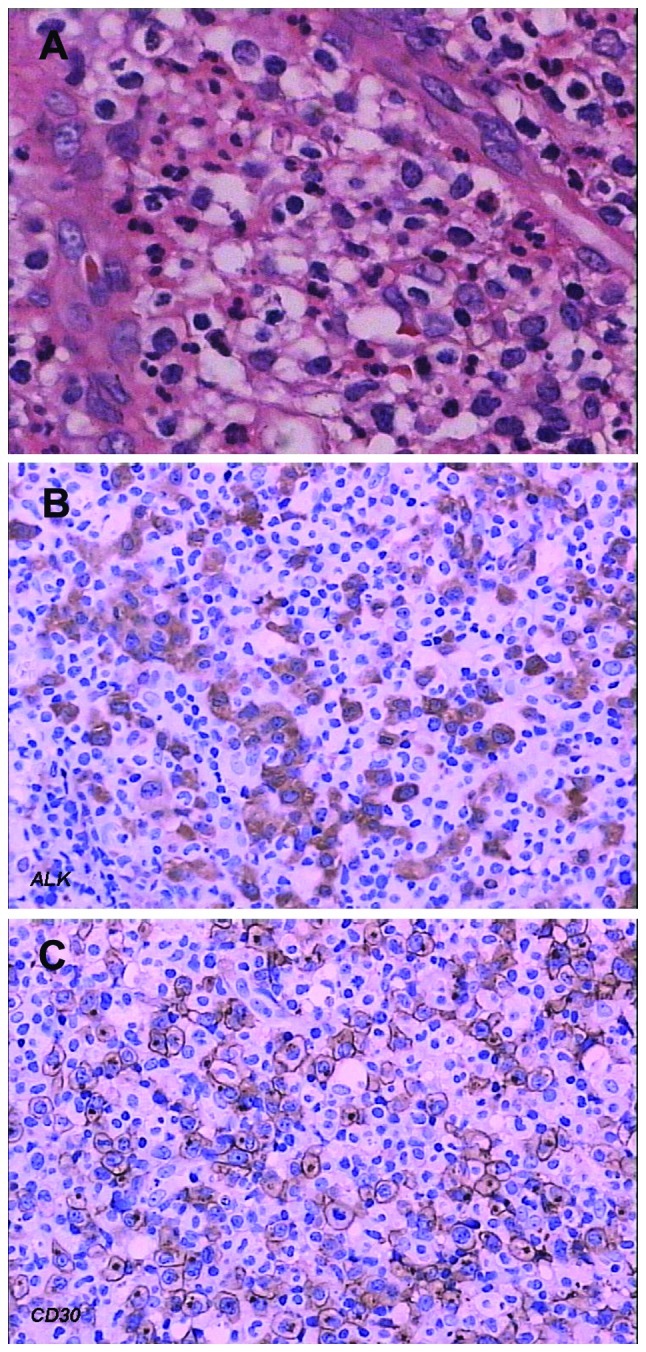
Morphological and immunophenotypical features of neutrophil-rich ALCL (case 15). (A) A number of neutrophils are admixed with large hallmark cells (hematoxylin and eosin staining; original magnification, ×400). (B) Immunostained for ALK. The neoplastic cells demonstrate a strong positivity in their cytoplasm. (C) Immunostained for CD30. The neoplastic cells demonstrate a strong positivity in their membrane. ALCL, anaplastic large cell lymphoma; ALK, anaplastic lymphoma kinase.

The patient with neutrophil-rich type ALCL presented fever and pleural effusions. Paraffin sections revealed that the polymorphous neoplastic cells with irregular nuclei dispersed. The neoplastic cells were intermixed with a large number of mature neutrophils and a local small abscess was observed (Fig. 2A). Immunohistochemical stain of neoplastic cells was positive for ALK and CD30 (Fig. 2B and C). B-cell markers, including CD79a and CD20 were negative in all cases.

### FCI results

**Figure 3.**
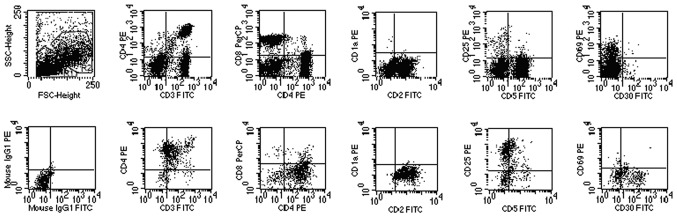
Flow cytometric analysis of lymph node biopsy specimen of ALCL case 6. Forward scatter (FSC) and side scatter (SSC) biparametric dot plot showing two lymphocytic populations (R1 and R2 regions). The cells in the R1 region with normal phenotype (CD2^+^, CD3^+^, CD5^+^, CD69_dim_, CD1a^−^, CD25^−^ and CD30^−^) and normal CD4/CD8 ratio were suggested as normal mature T cells by FCI (top panel). Flow cytometry detected a population of T lymphocytes (19.2%; R2 regions) with an abnormal phenotype (CD3_dim_, CD2^+^, CD4^+^, CD30^+^, CD25^+^, CD5^−^, CD69^−^, CD1a^−^ and CD8^−^; bottom panel) and high FSC/SSC, similar to monocytes and granulocytes, suggesting ALCL cell origin. ALCL, anaplastic large cell lymphoma; FCI, flow cytometry immunophenotyping.

**Figure 4.**
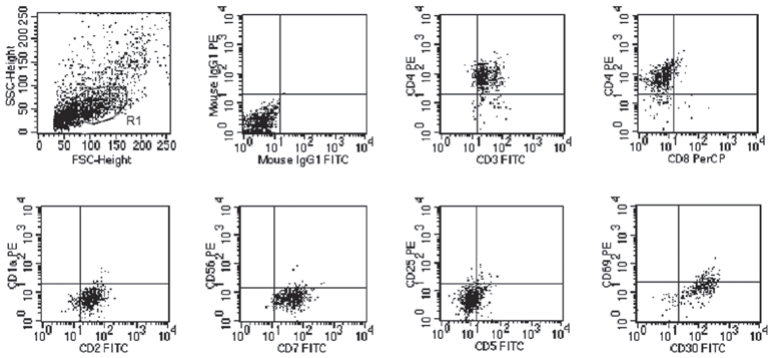
Flow cytometric analysis of lymph node biopsy specimen of ALCL case 9. The neoplastic cells are concentrated in the large lymphocyte region with an abnormal immunophenotype (CD3_dim_, CD2^+^, CD7^+^, CD4^+^, CD30^+^, CD5^−^, CD25^−^, CD56^−^, CD69^−^, CD1a^−^ and CD8^−^). ALCL, anaplastic large cell lymphoma.

Of the 15 cases with ALCL analyzed in this study, 13 (10 common, 2 lymphohistiocytic and 1 neutrophil-rich type) were diagnosed as ALCL with FCI. These 13 cases were CD30-positive and immunophenotypically aberrant with respect to expression of the T-cell antigens CD2, CD3, CD4, CD5 and CD7. CD30-positive neoplastic cells accounted for a small proportion of the total cells with FCI, with a median of 19.3% (range, 7.9–31.8%). The frequently expressed T-cell antigens were CD4 (84.6%), CD2 (76.9%), CD7 (61.5%), CD3 (53.8%) and CD5 (38.4%). Dim expression of CD3 was observed in ALCL cases compared with that in the background reactive T cells (median MFI, 293 vs. 95, P=0.018). CD25 was brightly expressed in the neoplastic cells in 10 of the 13 ALCL cases analyzed (76.9%); however, it was almost not expressed in background reactive T cells. CD45 was positive in all cases. CD8 and B-cell markers, including CD19, CD20, CD22, CD23, FMC7 and surface immunoglobulin were negative in all cases. In the majority of cases, the neoplastic cells demonstrated high forward and high side scatter, similar to monocytes or granulocytes on a dot plot (Fig. 3). In 3 common type cases, the neoplastic cells were concentrated in the large lymphocyte region (Fig. 4).

In the remaining 2 specimens with lymphohistiocytic type presenting extensive necrosis (cases 13 and 14), FCI revealed a low percentage (0.60 and 1.5%, respectively) of CD30-positive cells, thus FCI was not able to establish a diagnosis due to the relative rarity of the CD30-positive cells in the specimens.

**Figure 5.**
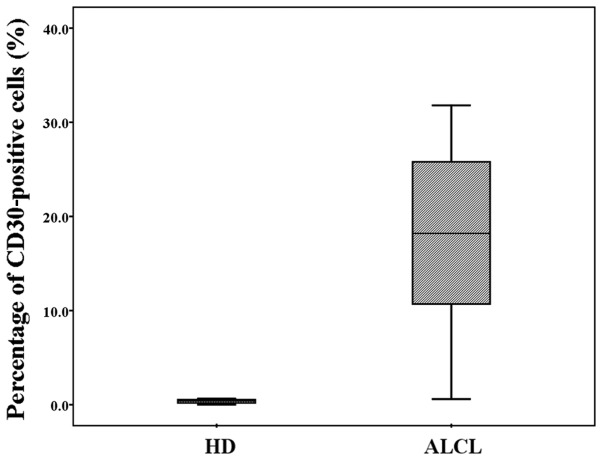
Comparison of the percentage of CD30-positive cells among patients with HD (n=7) and ALCL (n=15). The central horizontal line in the box is the median (50th percentile), the bottom and top of the box are the 25th and 75th percentiles. The whiskers extend from each end of the box to the 5th and 95th percentiles of the values, respectively. Outliers are data with values beyond the 5th and 95th percentiles. HD, Hodgkin lymphoma; ALCL, anaplastic large cell lymphoma.

In 99 cases of non-ALCL diseases, there were 27 cases of reactive hyperplasia, 4 cases of tuberculosis, 11 cases of necrotic lymphadenitis, 25 cases of B-NHL and 32 cases of T-LBL. FCI analysis did not show the presence of cells with coexpression of CD30 and T-cell antigens. In the remaining 7 cases with HD, FCI revealed a low percentage (median, 0.52% with a range of 0.01–0.65%) of CD30-positive cells with a dim expression, but did not establish a diagnosis for these cases due to the relative rarity of the CD30-positive cells in the specimens. The percentage of CD30-positive cells among patients with HD and ALCL is shown in Fig. 5.

In this study, the estimated sensitivity of FCI for diagnosing ALCL in lymph node samples was 86.7% (13/15 cases) with a specificity of 100%. When the specimens with necrosis were excluded from the analysis, the sensitivity increased to 100%, suggesting that FCI is a reliable approach for the diagnosis of ALCL when a neoplastic cell clone presents >5% cells with coexpression of CD30 and any of the T-cell antigens, identified by flow cytometry.

## Discussion

ALCL is a rare disease in children, accounting for 10–15% of all childhood NHL (11). The majority of ALCLs in children and adolescents are ALK-positive. In our cohort, the male to female ratio was 2:1. Previous studies demonstrated that there may be a male predominance, particularly in ALK(+) cases, in which the male to female ratio is ∼3:1 (12).

Histologically, several ALCL variants have been described. Morphological variants of ALCL include the following types: common, lymphohistiocytic, small cell and rare subforms, including neutrophil-rich types (13). Of these variants, the common, lymphohistiocytic and small cell types are the most frequently encountered. The ‘horseshoe’ or ‘wreath’ cell is considered the cytologic hallmark of this disease. Histological characteristics may have a high potential for future risk stratification and treatment (14).

Since ALCL demonstrates a broad morphological spectrum, definitive diagnosis and differential diagnosis of ALCL from other forms of lymphoma and reactive lymphadenopathy are difficult. Unlike other lymphomas, the tumor masses comprise normal reactive T cells and neoplastic cells in ALCL and in a number of cases only a careful search is likely to reveal the presence of neoplastic cells. Diagnosis of common type ALK(+) ALCL has become straightforward owing to the widespread availability of reliable anti-ALK antibodies. However, ALK(+) ALCL includes a morphological spectrum with small cell and lymphohistiocytic variants that represent ∼10–20% of cases and is easily confused with reactive lymphadenopathy (15). In our study, the paraffin sections of two cases of lymphohistiocytic ALCL demonstrated varying degrees of necrosis, which causes the misdiagnosis of inflammation. Scattered neoplastic cells were observed with a careful search and recognized by an immunohistochemical stain of CD30- and ALK-positive cells. In a neutrophil-rich type patient who was misdiagnosed with an inflammatory disease in a local hospital, the polymorphous neoplastic cells with irregular nuclei dispersed and were intermixed with a large number of mature neutrophils. CD30- and ALK-positive results suggest a diagnosis of ALCL in this case. The neutrophil-rich variant of anaplastic large cell lymphoma (NR-ALCL) is a rare type of NHL. Diagnosis by lymph node biopsy is difficult owing to the rarity of this tumor, its resemblance to HD and other NHL, its similarity to an infectious process and its occasional confusion with metastatic carcinoma and melanoma (16). Numerous ALCL cases present lymph node sclerosis and may be misdiagnosed as HD in which CD30 is also positive (17). As ALCL is a peripheral T-cell-derived malignancy, an immunohistochemical stain for T cell-specific markers, CD15, EMA and ALK protein may be useful in the differential diagnosis between ALCL and HD. In addition, the nodal ALCL should be differentiated from metastatic undifferentiated carcinoma, malignant melanoma, Langerhans cell histiocytosis and soft tissue sarcoma. Therefore, lymphadenopathy in children with abnormal cells in a background of inflammation should be considered as ALCL. Immunohistochemical staining of ALK and CD30 is a useful approach to confirm the diagnosis of ALCL.

Flow cytometry provides rapid analysis of multiple characteristics of separate cell populations based on their sizes, cytoplasmic characteristics and antigens expressed. Lymph node specimens are now routinely submitted for flow cytometric analysis in patients with suspected lymphoma and are considered to be standard practice in a number of centers. By comparison, relatively few studies have addressed the use of flow cytometry in the evaluation of ALCL. In the present study, we analysed 15 samples from patients with ALK(+) ALCL with FCI, 13 of which were consistent with pathological results. The sensitivity and specificity of FCI for diagnosing ALCL were 86.7 and 100%, respectively. The samples were CD30-positive and immunophenotyically aberrant with respect to T-cell antigen expression (CD2, CD3, CD4, CD5 and CD7), which is in concordance with the previous study (9). CD3 antigen was often expressed at a dim intensity compared with background normal T cells and such a marked contrast in the same FCI plot contributes to the diagnosis of ALCL. The frequent expression of CD25 in ALCL suggests that this antigen is a potentially useful marker in the immunophenotypic diagnosis of ALCL and a potential therapeutic target (18).

ALCL should also be differentiated from HD when diagnosed using FCI. Unlike ALCL in which the neoplastic cells dispersed in lymph node tissue, the Reed-Sternberg (RS) cells of HD usually scatter, where they constitute <1% and frequently <0.01% of the total cells (19). Therefore, routine analysis of 10,000 events by FCI is not able to bring a population that represents 0.01% of the total cells well within range of sensitivity of clinical cytometry, thus FCI is not able to establish a diagnosis in routine practice. By comparison, neoplastic cells have a relatively higher proportion of total cells in lymph node tissue with ALCL and are rapidly detected with FCI. We suggest that when CD45-, CD30- and T-cell marker-positive neoplastic cells constitute at least 5% of the total cells, a diagnosis of ALCL may be made using FCI.

Unlike other PTCL-NOS in which the neoplastic cells are usually small- to medium-sized, the majority of ALCL cells are large and show increased FSC and SSC. Therefore they are concentrated largely or entirely outside the lymphocyte gate, similar to monocytes or granulocytes. Conventional gating for lymphoma cells with low FSC and SSC may lead to false-negativity and careful analysis is required since the proportion of the aberrant population with a diagnostic value may be extremely small. Flexible gating strategies are important in the diagnosis of ALCL. In this study, 2 specimens (lymphohistiocytic type) with extensive necrosis were not confirmed as ALCL by flow cytometry due to the lack of analyzable cells present in the specimens. Similarly, Muzzafar *et al*(10) were unable to identify neoplastic cells by flow cytometry in 4 of 23 (17.4%) adults with ALCL. There are a number of technical factors and potential pitfalls that make ALCL diagnosis by flow cytometry particularly challenging. False-negative FCI results in ALCL may be due to the necrosis and apoptosis commonly associated with such tumors and to the fragility of the large atypical neoplastic cells, which may be easily disrupted during sample processing (20). Sampling issues may also be involved due to the neoplastic cells focally distributed in the lymph node (21). Clinical application of FCI for ALCL may be expanded, with the exception of diagnosing ALCL in lymph node biopsy specimens. Our previous study revealed that in pleural effusion from a case with ALCL, which was considered negative for ALCL by morphological examination, FCI detected a minor proportion (9.3%) of aberrant T-cell population with high FSC/SSC, a positive expression of CD4, CD7, CD2, CD45RO and CD30 and a negative expression of CD5 and CD69 (22). Moreover, Damm-Welk *et al* considered that FCI using antibodies against ALK and CD30, sensitively and specifically detects circulating ALCL cells in bone marrow or blood (23). Therefore, FCI holds a clear advantage over morphological examination in such circumstances.

In summary, ALK(+) ALCL is a distinct subset of NHL morphologically and immunophenotypically. FCI may be used as an adjunct to histopathological examination for reliable diagnosis of pediatric ALCL with high specificity and sensitivity. It is rapid and suitable for emergency situations, allowing for therapeutic decisions to be made promptly. However, flexible gating strategies and careful analysis are required to identify neoplastic cells with FCI.

## Figures and Tables

**Table I.** Morphological subforms and immunohistochemical results of 15 cases with ALCL.

Case no.	Gender	ALK	CD3	CD43	CD30	CD15	CD68	CD1a	EMA	EBV	Histological subtype
1	M	+	−	+	+	−	−	−	−	−	Common
2	M	+	+	+	+	−	−	−	−	−	Common
3	M	+	−	−	+	−	−	−	−	−	Common
4	M	+	+	−	+	−	−	−	+	−	Common
5	F	+	−	+	+	−	−	−	−	−	Common
6	M	+	+	−	+	−	−	−	+	−	Common
7	M	+	−	−	+	−	−	−	−	−	Common
8	M	+	+	+	+	−	−	−	−	−	Common
9	F	+	+	−	+	−	−	−	+	−	Common
10	M	+	−	−	+	−	−	−	−	−	Common
11	M	+	+	−	+	−	+	−	+	−	Lymphohistiocytic
12	M	+	+	+	+	+	+	−	+	−	Lymphohistiocytic
13	F	+	−	−	+	−	+	−	+	−	Lymphohistiocytic
14	F	+	+	+	+	−	+	−	+	−	Lymphohistiocytic
15	F	+	+	+	+	+	−	−	−	−	Neutrophil-rich

ALCL, anaplastic large cell lymphoma; ALK, anaplastic lymphoma kinase; EMA, epithelial membrane antigen; EBV, Epstein-Barr virus.
